# Association of preeclampsia with infant *APOL1* genotype in African Americans

**DOI:** 10.1186/s12881-020-01048-4

**Published:** 2020-05-20

**Authors:** Anna K. Miller, Timur Azhibekov, John F. O’Toole, John R. Sedor, Scott M. Williams, Raymond W. Redline, Leslie A. Bruggeman

**Affiliations:** 1 Department of Genetics and Genome Sciences, Case Western Reserve University School of Medicine, Cleveland, USA; 2 Division of Neonatology, Department of Pediatrics, Metro Health Medical Center, Case Western Reserve University School of Medicine, Cleveland, USA; 3 Departments of Inflammation and Immunity and Nephrology, Cleveland Clinic, Case Western Reserve University School of Medicine, Cleveland, USA; 4 Department of Physiology and Biophysics, Case Western Reserve University School of Medicine, Cleveland, USA; 5 Department of Population and Quantitative Health Sciences, Case Western Reserve University School of Medicine, Cleveland, USA; 6 Departments of Pathology and Reproductive Biology, University Hospitals, Case Western Reserve University School of Medicine, Cleveland, OH USA

**Keywords:** African American, Genetics, Pathology, Placenta, Preeclampsia

## Abstract

**Background:**

Black women in the United States and Africa are at an increased risk for preeclampsia. Allelic variants in the gene for apolipoprotein LI, *APOL1*, are found only in populations of African ancestry, and have been shown to contribute significant risk for kidney disease. Recent studies suggest these *APOL1* variants also may contribute risk for preeclampsia.

**Methods:**

The association of preeclampsia with carriage of *APOL1* risk alleles was evaluated in a case-control study of deliveries from black women at a single center in Cleveland, Ohio that included gross and histopathologic evaluations of placental tissues (395 cases and 282 controls). Using logistic regression models, associations between fetal *APOL1* genotype and preeclampsia were evaluated using several case definitions based on prematurity and severity of preeclampsia, with uncomplicated term pregnancies as controls. Associations between *APOL1* genotype and pathological features were also examined.

**Results:**

The infant *APOL1* genotype was significantly associated with preeclampsia in a dominant inheritance pattern with odds ratio of 1.41 (*P*=0.029, 95% CI 1.037, 1.926). Stratifying preeclampsia cases by preterm birth, significant associations were detected for both recessive (O.R.=1.70, *P*=0.038) and additive (O.R.=1.33, *P*=0.028) inheritance patterns. *APOL1* genotype, however, was not significantly associated with pathological changes or other perinatal observations.

**Conclusions:**

Preeclampsia appears to be another disease associated with *APOL1* variants, however, further studies are needed to increase confidence in the mode of inheritance. By understanding the association of *APOL1* variants with preeclampsia, genetic screening tests for *APOL1* may be useful to predict at-risk pregnancies and targeted interventions may be developed to improve pregnancy outcomes.

## Background

Preeclampsia (PE) is a common complication of pregnancy and occurs more frequently in women of African ancestry (reviewed in [1, 2]). PE prevalence ranges from 3-8% in developed countries and as high as 26% in sub-Saharan African countries, and African American women are three times more likely to die from PE compared to European Americans. The American College of Obstetricians and Gynecologists [3] defines PE as new onset hypertension of ≥140mmHg systolic or ≥90mmHg diastolic along with proteinuria or other end organ dysfunction. A separate class of PE is defined as severe PE with blood pressures ≥160/110mmHg and proteinuria or other end organ dysfunction. PE is a heterogeneous disorder, with differences in clinical presentation and outcomes for early onset PE (placental type) in which symptoms occur before 34 weeks gestation, and late onset PE (maternal type) in which symptoms occur ≥34 weeks gestation (reviewed in [4]).

Like PE, chronic kidney disease (CKD) also is more prevalent in African Americans, and the recent description of two polymorphisms in the *APOL1* gene has advanced our understanding of the increased risk for CKD in African Americans (reviewed in [5]). APOL1-associated CKD risk is inherited as a recessive trait, and a high risk genotype is any combination of the two *APOL1* risk alleles known as G1 (rs73885319, p.S342G and rs60910145, p.I384M) and G2 (rs71785313, p.NYK388K), with the common alleles at each site not associated with CKD referred to as G0. APOL1 is better known as the trypanolytic factor in human serum and provides innate immunity against *Trypanosoma brucei brucei* infection [6]. The G1 and G2 variants are more recent evolutionary adaptations that extend immune protection (in a dominant inheritance pattern) against *T. b. rhodesiense* and *T. b. gambiense* infections as causes of African sleeping sickness. The G1 and G2 variants are absent in individuals without recent African ancestry.

*APOL1* is expressed in several organs, most predominantly the liver, which secretes the abundant circulating APOL1 that kills trypanosomes. *APOL1* also is expressed (but not secreted) by several other tissues including the kidney [7, 8] and placenta (trophoblasts) [9–12]. The function of this intracellular APOL1 to homeostatic and pathogenic processes is not fully understood. Since a functional *APOL1* gene is present only in some primates, we created transgenic mice to model the expression of intracellular APOL1. These mice unexpectedly developed PE or eclamptic seizures and had small litter sizes from both fetal and neonatal deaths, both of which were independent of an underlying CKD [12]. The occurrence of PE/eclampsia was dependent on the *APOL1* genotype of the pup, not the dam, indicating transgenic *APOL1* expressed by the fetus or placenta triggered these phenotypes.

In humans, several studies have suggested *APOL1* expression may contribute to pregnancy complications including PE [13–15]. Although prior genome wide association studies have not identified *APOL1* risk alleles as candidates for PE, these studies had limitations such as excluding individuals of African ancestry, not examining informative markers for the *APOL1* variants, or only genotyping the mother and not the child [16–19]. In reports specifically evaluating *APOL1* risk alleles effects, association studies from pediatric cohorts found children with glomerular disease had a higher prevalence of preterm birth and low birth weight if they had two *APOL1* risk variants [20], although this association was not replicated in children without CKD [21]. A recent study of mother-child dyads from independent cohorts in New York and Tennessee associated PE (O.R.=1.84 and 1.92 respectively) with a recessive inheritance of *APOL1* risk variants in the child but not the mother [22].

To further establish association of PE with *APOL1* risk alleles in the infant, and to assess correlates with placental pathology, we examined an archival collection of placental tissues from black women in the Cleveland area. Genotyping of infant tissue confirmed an association of *APOL1* risk alleles with PE, although this association was most significant under a dominant inheritance pattern. Examining preterm PE cases separately, the association with *APOL1* genotype was significant in both recessive and additive models. Although the PE cases exhibited typical pathology consistent with a PE diagnosis and prematurity, there were no specific pathological features that correlated with *APOL1* genotype.

## Methods

### Study population

The Ohio March of Dimes biobank is a single center, ten year collection (2005-2014) of 8006 singleton pregnancies in which placentas, umbilical cords, and fetal membranes were collected for pathological evaluation, including normal term pregnancies. It excluded cases of congenital malformations, genetic disorders, and infections associated with congenital anomalies. The biobank is linked with a dataset (Supplemental Table 1) of maternal medical history, demographics, previous pregnancy history, and clinical data from the current pregnancy including early and late antenatal periods, intrapartum and birth data, and initial neonatal observations. In addition, gross placental measurements and standardized histopathologic diagnoses were recorded according to the Amsterdam Placenta Working Group definitions [23]. Since *APOL1* risk variants are only found in individuals of African ancestry, the study cohort was limited to women who self-identified as black (African American) and included both full term (≥37 weeks) and preterm (>20 to <37 weeks) spontaneous and elective deliveries. Controls were self-identified black women with full term pregnancies and no history of PE or other significant antenatal condition whose placentas were submitted to pathology for other indications. PE diagnosis and severity was specified by the submitting physician using criteria standardized by the American College of Obstetricians and Gynecologists [3]. PE severity was classified as either not-otherwise-specified (NOS) based on blood pressure ≥140 to <160mmHg systolic or ≥90 to <110 mmHg diastolic or severe for cases with blood pressure ≥160 mmHg systolic or ≥110 mmHg diastolic.

### Genotyping

Archived samples from the Ohio March of Dimes biobank were used for fetal genomic DNA extraction (Qiagen RecoverAll Total Nucleic Acid Isolation Kit) from two, 10μm sections of formalin-fixed, paraffin embedded umbilical cords. A TaqMan allele discrimination assay (ThermoFisher Scientific) was used to identify the two CKD-associated *APOL1* risk alleles as previously described for polymorphisms rs73885319 (representing the G1 allele) and a rs71785313 (G2 allele) [24].

### Statistical analysis

Summary statistics were performed by clinical category, comparing to term controls: all PE cases, preterm PE cases, term PE cases, term PE cases classified as NOS, term PE cases classified as severe. Summary statistics were performed for maternal risk factors, pathological features, and *APOL1* genotype. Unadjusted and adjusted logistic regression models were used to assess the association between *APOL1* variants and all PE cases and the subtypes described above. A forward stepwise selection procedure was used to determine the clinical/pathological variables that were included in adjusted models. For categories with a sample size of less than five cases with the scored feature, combined analyses were used or not done. *APOL1* genotype was examined for dominant, recessive, or additive inheritance models. The association between *APOL1* and pathological features were tested within clinical categories using a global chi-square and compared by dominant, recessive, or additive inheritance pattern. Pathological features were tested for association with *APOL1* by chi-square and ANOVA for categorical and continuous variables respectively. All analyses were performed in R version 3.5.1. The primary dataset generated and analyzed for this study is included as supplementary information (supplemental Primary Dataset).

## Results

The study population selected from the March of Dimes biobank consisted of 395 PE cases with 282 control pregnancies. Study inclusion/exclusion criteria are detailed in Supplemental Table 2; of note, HELLP syndrome (hemolysis, elevated liver enzymes, low platelet count) and comorbid conditions associated with PE risk such as gestational diabetes and pre-pregnancy hypertension were excluded. There were significant differences between controls and cases for maternal age and gravidity (Table 1). As expected for births complicated by PE, there were significant differences in gestational age, birth weight, and placenta weights. Both term and preterm cases had significantly lower birth weights. Placental weights were similar between controls and term cases, but significantly lower in preterm cases. Fetal/placental weight ratios, an indicator of placental efficiency, were significantly lower in both preterm and term cases.

**Table 1:** Demographics of study population and case subgroups, mean (SD)

	Controls	All cases		Case Term	Cases Preterm	Cases Term NOS	Cases Term severe
	*n* = 282	*n* = 395	P*	*n* = 204	*n* = 191	*n* = 58	*n* = 146
Maternal age, year	21.7 (4.4)	23.6 (5.5)	<0.001	23.4 (5.7)	23.9 (5.4)	23.6 (4.9)	23.3 (6.0)
Gravidity (median)	1	2	<0.001	2	2	2	2
Gestational age, week	39.4 (1.2)	36.4 (3.5)	<0.001	38.9 (1.2)	33.7 (3.2)	39.1 (1.2)	38.8 (1.2)
Birth weight, g	3146 (516)	2548 (762)	<0.001	2997 (525)	2008 (642)	3052 (474)	2977 (543)
Fetal/placental weight	7.7 (4.8)	6.6 (1.3)	0.001	7.0 (1.2)	6.0 (1.2)	6.8 (1.0)	7.1 (1.2)
Placental weight, g	451 (201)	385 (119)	<0.001	437 (91)	329 (121)	455 (78)	430 (95)

**Fig. 1:**
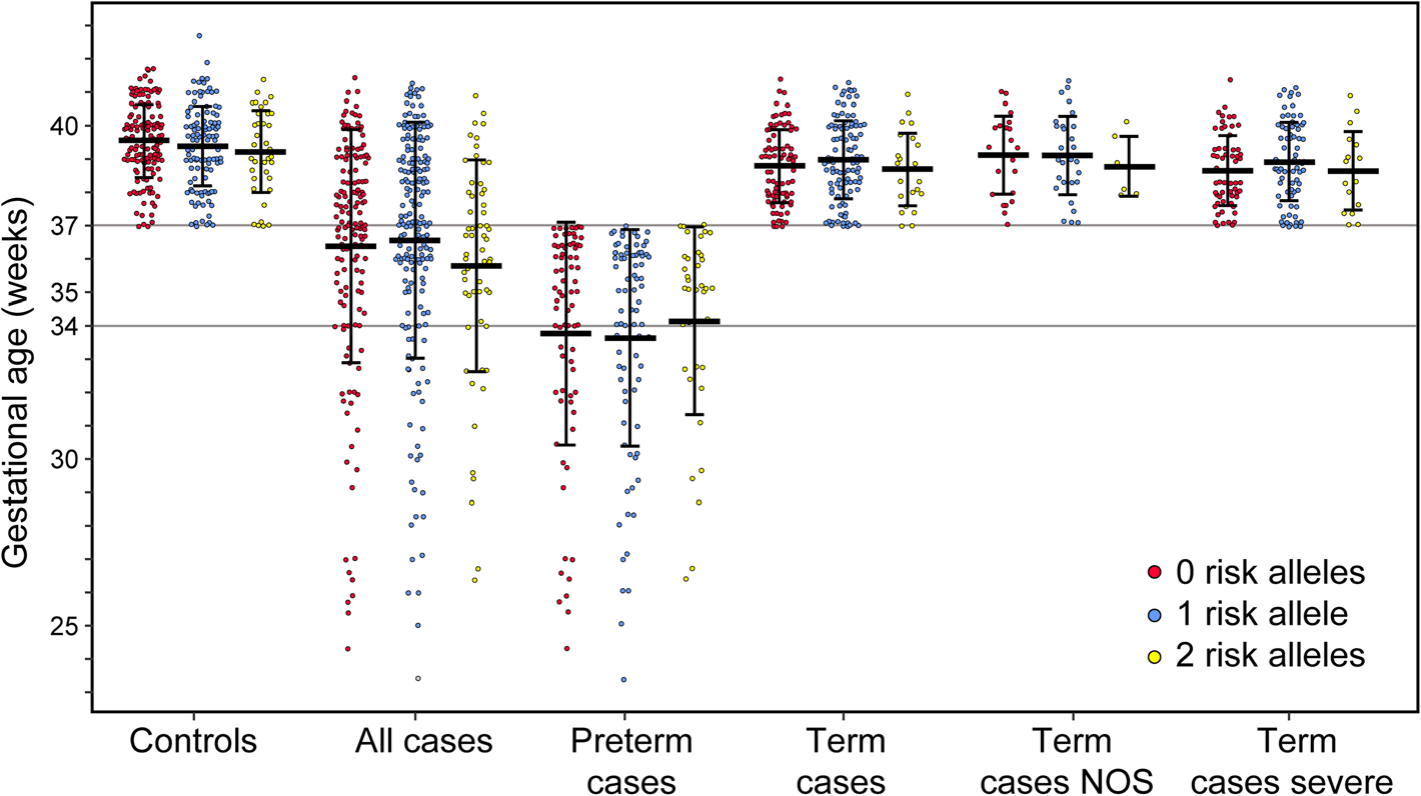
No difference in gestational age based on *APOL1* genotype. Preterm was defined as less than 37 weeks gestation, and early onset PE is before 34 weeks gestation. Although our cohort only recorded time of delivery and not onset of PE symptoms, all deliveries prior to 34 weeks would be from early onset PE. Controls versus all cases and preterm cases were significantly different (*P*<0.001), but there was no difference in gestation age within each case definition by *APOL1* genotype. Data are mean±SD

*Controls versus all cases. Gestational age data stratified by *APOL1* genotype are shown in Fig. 1.

### *APOL1* genotype and allele frequencies

*APOL1* allele and genotype frequencies from PE pregnancies were compared to uncomplicated term pregnancies using umbilical cord tissue, reflecting the infant’s genome. *APOL1* genotype frequencies did not deviate from Hardy-Weinberg equilibrium in controls or any case category (*P*>0.05). The control group genotype frequencies (Table 2) of 0, 1, or 2 risk alleles (47%, 40%, 13% respectively) were near reported genotype frequencies in African Americans of 40%, 46%, 14% [25]. Similarly, the control group allele frequencies (Table 2) for G0, G1 and G2 (67%, 23%, and 10% respectively) did not differ significantly from reported general population frequencies of 62-65%, 22-23%, and 13-15% (summarized in [24]). There was an enrichment of one risk allele and two risk allele genotypes in PE cases. These differences in *APOL1* genotype between cases and controls were analyzed by logistic regression models for recessive, additive, and dominant inheritance patterns.

**Table 2:** *APOL1* alleles and genotype frequencies

		Controls	All Cases	Cases Term	Cases Preterm	Cases Term NOS	Cases Term severe
**Allele Frequency**	G0	377 (67%)	488 (62%)	263 (64%)	225 (59%)	75 (65%)	188 (64%)
	G1	132 (23%)	203 (26%)	103 (25%)	100 (26%)	30 (26%)	73 (25%)
	G2	55 (10%)	99 (13%)	42 (11%)	57 (14%)	11 (9%)	31 (11%)
**Genotype Frequency**	0 risk allele	132 (47%)	153 (39%)	79 (39%)	72 (38%)	23 (40%)	58 (40%)
	1 risk allele	113 (40%)	182 (46%)	101 (50%)	81 (42%)	29 (50%)	72 (49%)
	2 risk allele	37 (13%)	60 (15%)	22 (11%)	38 (19%)	6 (10%)	16 (11%)

### Association between PE and *APOL1* genotype by logistic regression modeling

For regression modeling, pathological features were evaluated for potential adjustment as covariates. The presence and severity of placental pathology were compared between all case definitions with control pregnancies by chi-square analyses (Supplemental Table 3). PE cases (all, and stratified as term or preterm) were significant for the presence of villous infarcts. Infarction-hematomas (also known as round intraplacental hematomas), considered to represent intraplacental abruptions [26, 27], also were significantly more common in the preterm cases. The overall placental maturity based on the microscopic villous architecture (referred to as “villous architecture maturity”) was considered in conjunction with gestational age based on the term and preterm designations. The term cases were not significantly different than the control group. As expected, the preterm cases were more immature and significantly different than the controls. The differences in both infarction-hematomas and the villous architecture maturity in the preterm cases was the main contributor to the significant differences in the all cases group, as these were not significantly different in the term cases. All other pathological features were not significantly different between PE cases and controls.

In an unadjusted logistic regression model (Table 3), the association of the *APOL1* genotype with PE (all cases) was only significant in a dominant inheritance pattern with an O.R.=1.41. No other mode of inheritance was significant.

**Table 3:** PE association with fetal *APOL1* genotype: Unadjusted model

	Dominant model			Recessive model			Additive model		
	OR	95% CI	***P***	OR	95% CI	***P***	OR	95% CI	***P***
All Cases	1.41	1.037, 1.926	0.029	1.22	0.788, 1.923	0.373	1.23	0.990, 1.542	0.063
Term cases	1.38	0.961, 1.998	0.082	0.83	0.464, 1.442	0.507	1.13	0.869, 1.476	0.358
Preterm cases	1.44	0.994, 2.102	0.055	1.70	1.030, 2.801	0.038	1.33	1.033, 1.728	0.028
Term cases, NOS	1.36	0.768, 2.441	0.297	0.79	0.287, 1.844	0.611	1.10	0.725, 1.639	0.659
Term cases, Severe	1.39	0.930, 2.099	0.109	0.84	0.439, 1.548	0.588	1.14	0.854, 1.528	0.368

Three additional models were examined adjusting for pathological features and cohort characteristics that were different between cases and controls using a forward stepwise selection procedure. Model 1 (Supplemental Table 4A) adjusted for maternal age, Model 2 (Supplemental Table 4B) adjusted for maternal age and villous architecture maturity, and Model 3 (Supplemental Table 4C) adjusted for maternal age, villous architecture maturity, and gravidity. In all adjusted Models, the association between *APOL1* genotype and PE (all cases) remained significant for the dominant inheritance pattern with an O.R.=1.40 to 1.43.

When cases were stratified by prematurity or severity of PE, the significance of the *APOL1* genotype association varied by mode of inheritance. In all models except Model 1, preterm PE cases were significant for a recessive inheritance pattern with O.R.=1.70 to 1.76. In all models except Model 3, the preterm PE cases also were significant in an additive inheritance pattern (O.R.=1.32 to 1.35). For the term PE cases, no association was significant in all models and for any mode of inheritance, including when stratified by NOS or severe PE. In all adjusted models, inclusion of these variables had minimal effect on odds ratios.

### Association of *APOL1* genotype with pathology

Pathologic features also were analyzed by mode of inheritance of *APOL1* alleles (Supplemental Tables 5A and 5B). There were no features that associated with any inheritance pattern by case status. Gestational age, which was significantly different between cases and controls (Table 1), was not significantly different based on *APOL1* genotype within any case definition (Fig. 1). In addition, clinical observations and pathological features were analyzed for associations with the number of *APOL1* risk alleles (Supplemental Table 6). None of the clinical or pathological factors differed based on *APOL1* genotype alone.

## Discussion

Although the underlying causes for PE are multi-factorial, genetic factors have been recognized as likely contributors to PE risk. A recent report by Reidy *et al*. [22] genotyped *APOL1* risk variants in two geographically distinct cohorts and attributed PE risk with a recessive inheritance of *APOL1* risk alleles in the infant, but not the mother. Our observations are consistent with this report, however, the mode of inheritance in our study was most significant for dominant inheritance in all cases. Only our preterm PE cases were significant for a recessive inheritance pattern, at a similar odds ratio as reported in the Reidy *et al*. study. We did not find an association between *APOL1* genotype and the gestational age at time of birth, severity of PE, or changes in pathology which also is consistent with Reidy *et al*. study. Our cohort excluded cases of HELLP syndrome and history of diabetes or hypertension, and morbid obesity. The Reidy *et al*. cohorts did not exclude these conditions, however their *APOL1* genotype associations were not influenced by these variables.

Our study did not observe a significant association of *APOL1* genotype with any scored pathologic feature at delivery. This lack of association could indicate the *APOL1*-dependent mechanism is not directly linked to decreased endovascular invasion by trophoblasts and inadequate spiral arteriolar remodeling, and that the causal event mediated by *APOL1* is not limited to the subgroup of PE associated with severe placental maternal vascular malperfusion (i.e. ischemic placenta). However, this may not exclude a unique role for *APOL1* in inducing a novel process in trophoblasts earlier in pregnancy, which then becomes a critical factor for the failed trophoblast invasion, subsequently initiating the typical cascade of events from placental ischemia to PE. The clinical heterogeneity of PE is related to both placental gene expression and placental histopathology [28, 29], and pathogenesis is multifactorial and frequently dependent on the maternal response to reach a threshold for the toxic systemic vascular reaction [30]. Our *APOL1* mouse models may provide an experimental platform to assess these issues throughout pregnancy, including the timing of trophoblast *APOL1* expression, characterization of placental gene expression patterns, maldevelopment of typical placental structures, and systemic maternal responses.

Overall, our observations are consistent with the original observation in Reidy *et al*. that fetal *APOL1* risk alleles contribute to PE risk, however, additional questions remain, specifically regarding the inheritance pattern when considering gestational age. Different genetic models apply to the various disease susceptibilities associate with *APOL1* risk alleles (i.e. CKD risk is recessive, protection against trypanosomiasis is dominant), thus additional investigations are needed in both African American and African populations to establish the inheritance pattern for PE. It is possible there is a phenotypic difference in PE that occurs prior to 37 weeks gestation. In addition, it is possible there is a phenotypic difference with the carriage of the G1 versus G2 alleles. Since we were unable to genotype the mothers, another aspect of this study that could not be addressed is a possible discordance of *APOL1* alleles between mother and infant as a key part of the genotypic risk.

Some limitations of our study are similar to the Reidy *et al*. report, including the use of cohorts from other investigations that was not originally designed to examine the effects of maternal and infant *APOL1* genotypes on PE. As such, additional prospective studies are needed to help clarify the mode of inheritance and to evaluate the mechanism driven by *APOL1* expression in either the fetus or placenta. Due to the use of archived specimens, our study did not have suitable and sufficient tissue for maternal genotyping and was unable to confirm the lack of association with the maternal *APOL1* genotype. For the same reason, we were unable to genotype ancestry informative markers to eliminate possible population substructure as a confounder. In addition, we cannot rule out the *APOL1* alleles are only markers in linkage disequilibrium with the true causal genetic variant.

## Conclusions

This is a confirmatory report attributing a significant risk for PE associated with the fetal carriage of *APOL1* variant alleles in African American women. Considering the high prevalence of these alleles in many African and African diaspora communities, future studies of both African American and African women will be useful in addressing a potentially significant cause of PE worldwide. Development of *APOL1* genotyping strategies may be clinically useful in identifying mothers at risk for PE.

## Supplementary information

**Additional file 1: Table 1.** Summary of clinical information and pathological categories in the Ohio March of Dimes biobank. **Table 2.** Summary of inclusion/exclusion criteria. **Table 3.** Pathological variable summary statistics (chi square test). **Table 4A.** PE association with fetal *APOL1* genotype: Adjusted Model 1. **Table 4B.** PE association with fetal *APOL1* genotype: Adjusted Model 2. **Table 4C.** PE association with fetal *APOL1* genotype: Adjusted Model 3. **Table 5A.** Genotype comparisons for global chi square interactions. **Table 5B.** Association of *APOL1* inheritance pattern with pathologic features (global chi square, *P* values). **Table 6.** Pathologic features by *APOL1* genotype.

## Data Availability

All data generated and analyzed during this study are included in this published article and its supplementary information files.

## Abbreviations

APOL1: Apolipoprotein 1
CKD: Chronic kidney disease
HELLP: Hemolysis, elevated liver enzymes, low platelet count
NOS: Not-otherwise-specified
PE: Preeclampsia
