# Supplementary material for: Association of preeclampsia with infant APOL1 genotype in African Americans
Source: BMC Med Genet. 2020 May 20;21:110. doi: 10.1186/s12881-020-01048-4 (PMC7238556; doi:10.1186/s12881-020-01048-4)
Supplement: Supplementary file 1 — Additional file 1: Table 1. Summary of clinical information and pathological categories in the Ohio March of Dimes biobank. Table 2. Summary of inclusion/exclusion criteria. Table 3. Pathological variable summary statistics (chi square test). Table 4A. PE association with fetal APOL1 genotype: Adjusted Model 1. Table 4B. PE association with fetal APOL1 genotype: Adjusted Model 2. Table 4C. PE association with fetal APOL1 genotype: Adjusted Model 3. Table 5A. Genotype comparisons for global chi square interactions. Table 5B. Association of APOL1 inheritance pattern with pathologic features (global chi square, P values). Table 6. Pathologic features by APOL1 genotype. [file 12881_2020_1048_MOESM1_ESM.pdf]

Supplemental Information

Supplemental Table 1. Summary of clinical information and pathological categories in the Ohio March of Dimes biobank.

Supplemental Table 2. Summary of inclusion/exclusion criteria.

Supplemental Table 3. Pathological variable summary statistics (chi square test).

Supplemental Table 4A. PE association with fetal *APOL1* genotype: Adjusted Model 1.

Supplemental Table 4B. PE association with fetal *APOL1* genotype: Adjusted Model 2.

Supplemental Table 4C. PE association with fetal *APOL1* genotype: Adjusted Model 3.

Supplemental Table 5A. Genotype comparisons for global chi square interactions.

Supplemental Table 5B. Association of *APOL1* inheritance pattern with pathologic features (global chi square, *P* values).

Supplemental Table 6. Pathologic features by *APOL1* genotype.

SUPPLEMENTAL TABLE 1. Summary of clinical information and pathological categories in the Ohio March of Dimes biobank.

Maternal demographics	Maternal co-morbidities	Antenatal condition	Current pregnancy	Current pregnancy condition
race	chronic hypertension	substance abuse	gestational age	pre-eclampsia (NOS, severe)
maternal age	diabetes	smoking	elective delivery	HELLP syndrome
prior full term deliveries	gestational diabetes	alcohol use	Apgar score	fetal growth restriction
prior preterm deliveries	obesity	STDs	birth weight	oligo/polyhydramnios
gravidity	history DVTs	anemia	neonate sex	cervical insufficiency
prior living children	coagulation disorder		mother death	cervical cerclage
repeat pregnancy loss	hyperthyroidism		neonate death	history vaginal bleeding
	asthma			placenta previa
	IVF/artificial reproduction			membrane rupture
				stat delivery
				maternal fever
**Placental gross observations**		**Placental histopathology (scored low grade or high grade)**		
placental weight		villous architecture maturity		
placental diameter		acute chorioamnionitis		
placental anomalies		meconium-related changes		
umbilical cord length and insertion		maternal vascular malperfusion		
umbilical cord anomalies		marginal abruption		
circumvallate membrane insertion		fetal vascular malperfusion		
		villitis of unknown etiology		
		fetal stromal vascular maldevelopment		
		perivillous fibrin(oid) deposition		

SUPPLEMENTAL TABLE 2: Summary of inclusion/exclusion criteria.

	Inclusions	Exclusions
**For all cases and controls**	African American<br>Spontaneous delivery<br>Elective delivery	HELLP syndrome<br>Pregestational diabetes<br>Gestational diabetes<br>Chronic hypertension<br>Intrauterine fetal death<br>$1^{st}/2^{nd}$ trimester STD<br>Antenatal substance abuse
**Case 1**: PE, Severe term	PE, Severe<br>≥ 37 weeks	PE, NOS
**Case 2**: PE, NOS term	PE, NOS<br>≥ 37 weeks	PE, Severe
**Case 3**: PE, preterm	PE, Severe and NOS<br>>20 to < 37 weeks	
**Control**	≥ 37 weeks	PE, NOS & Severe<br>Gestational hypertension<br>Other maternal co-morbidities<br>Other antenatal condition<br>Other current pregnancy condition<br>Prior preterm birth<br>Repeat pregnancy loss<br>Maternal or neonate death

*****Clinical definitions extracted from medical record, not ICD-9 coding.

PE, preeclampsia; NOS, not-otherwise-specified.

SUPPLEMENTAL TABLE 3: Pathological variable summary statistics (chi square test).

	Villous infarcts, % (#)				Infarction hematomas, % (#)				Villous architecture maturity, % (#)			
	None	Single	Multiple	*P*	None	Single	Multiple	*P*	Mature	Slightly Immature	Immature	*P*
Controls	93 (262)	6 (18)	1 (2)		99 (280)	0.5 (1)	0.5 (1)		71 (195)	27 (75)	2 (6)	
Cases, all	75 (297)	13 (50)	12 (48)	<0.001	96 (380)	3 (10)	1 (5)	0.039	56 (223)	39 (153)	5 (19)	<0.001
Cases, preterm	64 (123)	19 (36)	17 (32)	<0.001	95 (181)	4 (7)	1 (3)	0.008	47 (91)	47 (89)	6 (11)	<0.001
Cases, term	85 (174)	7 (14)	8 (16)	<0.001	98 (199)	1 (3)	1 (2)	0.276	65 (132)	31 (64)	4 (8)	0.279

Other pathologic features examined that were not significant included:

1. Maternal vascular malperfusion
2. Focally increased syncytial knots and fibrin with agglutination
3. Mural hypertrophy of decidual arterioles
4. Villitis of unknown etiology
5. Fetal stromal vascular maldevelopment
6. Fetal vascular malperfusion
7. Placental weight Z score

SUPPLEMENTAL TABLE 4A: PE association with fetal *APOL1* genotype: ADJUSTED MODEL 1

	Dominant model			Recessive model			Additive model		
	**OR**	**95% CI**	***P***	**OR**	**95% CI**	***P***	**OR**	**95% CI**	***P***
All Cases	1.42	1.038, 1.949	0.029	1.18	0.758, 1.872	0.463	1.23	0.982, 1.540	0.073
Term cases	1.42	0.978, 2.055	0.067	0.85	0.475, 1.477	0.559	1.14	0.871, 1.489	0.343
Preterm cases	1.48	1.013, 2.184	0.044	1.63	0.978, 2.721	0.060	1.35	1.039, 1.757	0.025
Term cases, NOS	1.43	0.800, 2.586	0.234	0.82	0.298, 1.941	0.679	1.14	0.747, 1.708	0.546
Term cases, Severe	1.44	0.955, 2.179	0.084	0.86	0.455, 1.589	0.647	1.15	0.857, 1.542	0.350

*adjusted for maternal age

SUPPLEMENTAL TABLE 4B: PE association with fetal *APOL1* genotype: ADJUSTED MODEL 2*

	Dominant model			Recessive model			Additive model		
	**OR**	**95% CI**	***P***	**OR**	**95% CI**	***P***	**OR**	**95% CI**	***P***
All Cases	1.43	1.041, 1.969	0.027	1.19	0.759, 1.895	0.448	1.24	0.989, 1.560	0.064
Term cases	1.43	0.984, 2.073	0.062	0.83	0.462, 1.460	0.522	1.16	0.883, 1.513	0.294
Preterm cases	1.48	0.999, 2.198	0.052	1.71	1.014, 2.905	0.044	1.35	1.035, 1.779	0.028
Term cases, NOS	1.43	0.801, 2.606	0.231	0.86	0.310, 2.039	0.749	1.15	0.755, 1.742	0.504
Term cases, Severe	1.45	0.960, 2.195	0.079	0.83	0.430, 1.547	0.570	1.17	0.868, 1.567	0.305

*adjusted for maternal age and villous architecture maturity

SUPPLEMENTAL TABLE 4C: PE association with fetal *APOL1* genotype: ADJUSTED MODEL 3*

	Dominant model			Recessive model			Additive model		
	**OR**	**95% CI**	***P***	**OR**	**95% CI**	***P***	**OR**	**95% CI**	***P***
All Cases	1.40	1.012, 1.940	0.042	1.17	0.737, 1.881	0.507	1.22	0.966, 1.540	0.096
Term cases	1.42	0.976, 2.065	0.068	0.82	0.454, 1.450	0.499	1.15	0.876, 1.508	0.316
Preterm cases	1.39	0.918, 2.121	0.121	1.76	1.007, 3.071	0.046	1.32	0.991, 1.765	0.058
Term cases, NOS	1.45	0.806, 2.665	0.219	0.87	0.312, 2.103	0.779	1.17	0.760, 1.778	0.476
Term cases, Severe	1.42	0.942, 2.160	0.096	0.81	0.417, 1.515	0.521	1.15	0.854, 1.547	0.352

*adjusted for maternal age, villous architecture maturity, and gravidity

Supplemental Table 5A. Genotype comparisons for global chi square interactions.

Inheritance pattern	Genotype 1	Genotype 2
Dominant	G0G0	G0G1 or G0G2
Recessive	G0G0	G1G2 or G1G1 or G2G2
Additive	G0G1 or G0G2	G1G2 or G1G1 or G2G2

Supplemental Table 5B. Association of *APOL1* inheritance pattern with pathologic features (global chi square, *P* values).

	Controls	Cases Term	Cases Preterm	Cases Term NOS	Cases Term Severe
Maternal Vascular Malperfusion	0.98	0.98	0.82	0.19	0.98
Fetal Vascular Malperfusion	0.72	*	0.89	*	0.90
Villitis of Unknown Etiology	0.81	*	0.88	*	0.46
Fetal Stromal Vascular Maldevelopment	0.44	0.97	0.15	0.66	0.83
Placental Weight Z Score	0.18	0.17	0.17	0.25	0.36
Fetoplacental Weight Ratio	0.88	0.25	0.75	0.99	0.28
Umbilical Cord Length	0.10	0.76	0.76	0.43	0.34
Villous Infarcts	0.35	0.81	0.99	0.90	0.89
Villous Infarction-Hematomas	0.80	0.75	0.73	*	0.80
Histological Feature of Abruptio Placenta	*	0.79	0.65	*	*
Mural Hypertrophy of Decidual Arterioles	0.68	0.40	0.49	0.53	0.56
Acute Atherosis of Decidual Arteries/Arterioles	*	0.47	0.65	0.67	0.28
Focally Increased Syncytial Knots and Fibrin with Agglutination	0.86	0.82	0.82	0.38	0.95

* Not analyzed due to insufficient number of cases with scored feature (small sample size).

Supplemental Table 6. Pathologic feature by *APOL1* genotype (none significant).

	Control			All cases			Term cases severe			Term cases NOS			Preterm cases		
**# risk alleles**	0	1	2	0	1	2	0	1	2	0	1	2	0	1	2
**N**	132	113	37	81	101	22	58	72	16	23	29	6	72	81	38
**Villous Architecture Maturity**															
Mature	96 (72.7%)	75(66.4%)	30(81.1%)	50 (61.7%)	67(66.3%)	15(68.2%)	36 (62.1%)	49(68.1%)	12(75.0%)	14 (60.9%)	18(62.1%)	3(50.0%)	34(47.2%)	41(50.6%)	16(42.1%)
Slightly immature	32 (24.2%)	36(31.9%)	7(18.9%)	27 (33.3%)	30(29.7%)	7(31.8%)	19 (32.7%)	20(27.8%)	4(25.0%)	8 (34.8%)	10(34.5%)	3(50.0%)	33(45.8%)	37(45.7%)	19(50.0%)
Immature	4 (3.0%)	2(1.8%)	0(0%)	4 (4.9%)	4(4.0%)	0(0%)	3 (5.2%)	3(4.1%)	0 (0%)	1 (4.3%)	1(3.4%)	0(0%)	5(7.0%)	3(3.7%)	3(7.9%)
**Maternal Vascular Malperfusion**															
None	116(87.9%)	102(90.3%)	32(86.5%)	52(64.2%)	69(68.3%)	16(72.7%)	36(62.1%)	42(58.3%)	11(68.8%)	16(69.6%)	27(93.1%)	5(83.3%)	32(44.4%)	36(44.4%)	16(42.1%)
Low Grade	10(7.6%)	7(6.2%)	4(10.8%)	20(24.7%)	22(21.8%)	3(13.6%)	13(22.4%)	20(27.8%)	3(18.7.%)	7(30.4%)	2(6.9%)	0(0%)	14(19.4%)	11(13.6%)	10(26.3%)
High Grade	6(4.5%)	4(3.5%)	1(2.7%)	9(11.1%)	10(9.9%)	3(13.6%)	9(15.5%)	10(13.9%)	2(12.5%)	0(0%)	0(0%)	1(16.7%)	26(36.1%)	34(42.0%)	12(31.6%)
**Villous infarcts**															
None	126(95.4%)	101(89.4%)	35(94.6%)	66(81.5%)	88(87.1%)	20(90.9%)	46(79.3%)	61(84.7%)	15(93.8%)	20(87.0%)	27(93.1%)	5(83.3%)	44(61.1%)	54(66.7%)	25(65.8%)
Single	5(3.8%)	12(10.6%)	1(2.7%)	7(8.6%)	7(6.9%)	0 (0%)	5(8.6%)	6(8.3%)	0(0%)	2(8.6%)	1(3.5%)	0(0%)	15(20.8%)	14(17.3%)	7(18.4%)
Multiple	1(0.8%)	0(0%)	1(2.7%)	8(9.9%)	6(5.9%)	2(9.1%)	7(12.1%)	5(6.9%)	1(6.2%)	1(4.4%)	1(3.5%)	1(16.7%)	13(18.1%)	13(16.1%)	6(15.9%)
**Focally Increased Syncytial Knots and Fibrin with Agglutionation**															
None	116(87.9)	99(87.6%)	30(81.1%)	45(55.6%)	59(58.4%)	14(63.6%)	30(51.7%)	35(48.6%)	8(50.0%)	15(65.2%)	24(82.8%)	6(100%)	29(39.7%)	34(42.0%)	14(37.8%)
Present	16(12.1%)	14(12.4%)	7(18.9%)	35(43.2%)	42(41.6%)	8(36.4%)	28(48.3%)	37(51.4%)	8(50.0%)	8(34.8%)	5(17.2%)	0(0%)	44(60.3%)	47(58.0%)	23(62.2%)
**Infarction-hematomas**															
None	130(98.5%)	113(100%)	37(100%)	80(98.8%)	98(97.0%)	21(95.5%)	57(98.3%)	69(95.8%)	16(100.0%)	23(100%)	29(100%)	5(83.3%)	66(91.7%)	78(96.3%)	37(97.4%)
Single	1(0.8%)	0(0%)	0(0%)	1(1.2%)	1(1.0%)	1(4.5%)	1(1.7%)	1(1.4%)	0(0%)	0(0%)	0(0%)	1(16.7%)	5(6.9%)	2(6.2%)	0(0%)
Multiple	1(0.8%)	0(0%)	0(0%)	0(0%)	2(2.0%)	0(0%)	0(0%)	2(2.8%)	0(0%)	0(0%)	0(0%)	0(0%)	1(1.4%)	1(1.2%)	1(2.6%)
**Histological Feature of Abruptio Placenta**															
None	132(100%)	113(100%)	37(100%)	79(97.5%)	97(96.0%)	22(100.0%)	56(96.5%)	68(94.4%)	16(100.0%)	23(100%)	29(100%)	6(100%)	70(97.2%)	76(93.8%)	37(97.4%)
Present	0(0%)	0(0%)	0(0%)	2(2.5%)	4(4.0%)	0(0%)	2(3.5%)	4(5.6%)	0(0%)	0(0%)	0(0%)	0(0%)	2(2.8%)	5(6.2%)	1(2.6%)
**Mural Hypertrophy of Decidual Arterioles**															
None	130(98.5%)	109(96.5%)	36(97.3%)	67 (82.7%)	91(90.1%)	18(81.8%)	48(82.8%)	64(88.9%)	12(75.0%)	19(82.6%)	27(93.1%)	6(100%)	56(77.8%)	57(70.4%)	32(84.2%)
Present	2(1.5%)	4(3.5%)	1(2.7%)	14(17.3%)	10(9.9%)	4(18.2%)	10(17.2%)	8(11.1%)	4(25.0%)	4(17.4%)	2(6.9%)	0(0%)	16(22.2%)	24(29.6%)	6(15.8%)
**Histological Feature of Abruptio Placenta**															
None	132(100%)	113(100%)	37(100%)	75(92.6%)	98(97.0%)	21(95.5%)	52(89.7%)	70(97.2%)	15(93.8%)	23(100%)	28(96.6%)	6(100%)	56(77.8%)	59(72.8%)	32(84.2%)
Present	0(0%)	0(0%)	0(0%)	6(7.4%)	3(3.0%)	1(4.5%)	6(10.3%)	2(2.8%)	1(6.2%)	0(0%)	1(3.4%)	0(0%)	16(22.2%)	22(27.2%)	6(15.8%)
**Fetal Vascular Malperfusin**															
None	116(87.9%)	105(92.9%)	34(91.9%)	75(92.6%)	87(86.1%)	18(81.8%)	54(93.1%)	65(90.3%)	13(81.3%)	21(91.3%)	22(75.9%)	5(83.3%)	66(91.7%)	73(90.1%)	36(94.7%)
Low Grade	15(11.3%)	7(6.2%)	2(5.4%)	6(7.4%)	13(12.9%)	3(13.6%)	4(6.9%)	6(8.3%)	2(12.5%)	2(8.7%)	7(24.1%)	1(16.7%)	5(6.9%)	8(9.9%)	2(5.3%)
High Grade	1(0.8%)	1(0.9%)	1(2.7%)	0(0%)	1(1.0%)	1(4.6%)	0(0%)	1(1.4%)	1(6.2%)	0(0%)	0(0%)	0(0%)	1(1.4%)	0(0%)	0(0%)
**Villitis of Unknown Etiology**															
None	126(95.5%)	107(94.7%)	35(13.6%)	74(91.4%)	100(99.0%)	20(90.9%)	53(91.4%)	71(98.6%)	14(87.5%)	21(91.3%)	29(100%)	6(100%)	65(90.3%)	77(95.1%)	35(92.1%)
Low Grade	2(1.5%)	4(3.5%)	2(5.4%)	3(3.7%)	0(0%)	1(4.5%)	3(5.2%)	0(0%)	1(6.2%)	0(0%)	0(0%)	0(0%)	2(2.8%)	2(6.2%)	1(2.6%)
High Grade	4(3.0%)	2(1.8%)	0(0%)	4(4.9%)	1(1.0%)	1(4.6%)	2(3.4%)	1(1.4%)	1(6.2%)	2(8.7%)	0(0%)	0(0%)	5(6.9%)	2(6.2%)	2(5.3%)
**Fetal Stromal Vascular Maldevelopment**															
None	118(89.4%)	103(91.1%)	36(97.3%)	72(88.9%)	90(89.1%)	22(100.0%)	51(87.9%)	65(90.3%)	16(100.0%)	21(91.3%)	25(86.2%)	6(100.%)	67(93.1%)	80(98.8%)	32(84.2%)
Low Grade	10(7.6%)	10(8.9%)	1(2.7%)	8(9.9%)	10(9.9%)	0(0%)	7(12.1%)	6(8.3%)	0(0%)	1(4.4%)	4(13.8%)	0(0%)	5(6.9%)	0(0%)	6(15.8%)
High Grade	4(3.0%)	0(0%)	0(0%)	1(1.2%)	1(1.0%)	0(0%)	0(0%)	1(1.4%)	0(0%)	1(4.4%)	0(0%)	0(0%)	0(0%)	1(1.2%)	0(0%)
**Placental Weight (mean Z score)**															
	-0.231	0.144	-0.186	-0.227	-0.224	-0.042	-0.269	-0.061	-0.219	-0.120	0.068	0.340	-0.218	-0.381	-0.423
